# Genetic diversity and trait genomic prediction in a pea diversity panel

**DOI:** 10.1186/s12864-015-1266-1

**Published:** 2015-02-21

**Authors:** Judith Burstin, Pauline Salloignon, Marianne Chabert-Martinello, Jean-Bernard Magnin-Robert, Mathieu Siol, Françoise Jacquin, Aurélie Chauveau, Caroline Pont, Grégoire Aubert, Catherine Delaitre, Caroline Truntzer, Gérard Duc

**Affiliations:** UMR1347, Agroecology, INRA, 17 rue de Sully, Dijon Cedex, 21065 France; Clinical and Innovation Proteomic Platform (CLIPP), CHU Dijon, Université de Bourgogne, 1 rue du Professeur Marion, Dijon, 21000 France; Present address: US EPGV, IG-CEA, Centre National de Génotypage, 2 rue Gaston Crémieux, Evry Cedex, 91057 France; UMR GDEC, Plateforme Gentyane, Clermont Ferrand, 63100 France

## Abstract

**Background:**

Pea (*Pisum sativum L.*), a major pulse crop grown for its protein-rich seeds, is an important component of agroecological cropping systems in diverse regions of the world. New breeding challenges imposed by global climate change and new regulations urge pea breeders to undertake more efficient methods of selection and better take advantage of the large genetic diversity present in the *Pisum sativum* genepool. Diversity studies conducted so far in pea used Simple Sequence Repeat (SSR) and Retrotransposon Based Insertion Polymorphism (RBIP) markers. Recently, SNP marker panels have been developed that will be useful for genetic diversity assessment and marker-assisted selection.

**Results:**

A collection of diverse pea accessions, including landraces and cultivars of garden, field or fodder peas as well as wild peas was characterised at the molecular level using newly developed SNP markers, as well as SSR markers and RBIP markers. The three types of markers were used to describe the structure of the collection and revealed different pictures of the genetic diversity among the collection. SSR showed the fastest rate of evolution and RBIP the slowest rate of evolution, pointing to their contrasted mode of evolution. SNP markers were then used to predict phenotypes -the date of flowering (BegFlo), the number of seeds per plant (Nseed) and thousand seed weight (TSW)- that were recorded for the collection. Different statistical methods were tested including the LASSO (Least Absolute Shrinkage ans Selection Operator), PLS (Partial Least Squares), SPLS (Sparse Partial Least Squares), Bayes A, Bayes B and GBLUP (Genomic Best Linear Unbiased Prediction) methods and the structure of the collection was taken into account in the prediction. Despite a limited number of 331 markers used for prediction, TSW was reliably predicted.

**Conclusion:**

The development of marker assisted selection has not reached its full potential in pea until now. This paper shows that the high-throughput SNP arrays that are being developed will most probably allow for a more efficient selection in this species.

**Electronic supplementary material:**

The online version of this article (doi:10.1186/s12864-015-1266-1) contains supplementary material, which is available to authorized users.

## Background

Pea (*Pisum sativum L.*) is a major pulse crop, with 9.8 million tonnes of dry seeds produced worldwide in 2012 [1]. This production is distributed in many temperate regions of the world with 3.4 million in Europe, 3.3 million in North America and 2 millions in Asia. As a member of the large family of legumes, pea presents both interesting biological features and attractive ecological services [2]. Thanks to the symbiosis it establishes with atmospheric-nitrogen fixing *Rhizobacteria*, pea crops do not require nitrogen fertilizer inputs and provide nitrogen to the following crop. Furthermore, pea seeds as well as leaves are a good source of plant proteins for human and animal nutrition. While facing new challenges such as the need for resistance to biotic and abiotic stresses, and for new seed quality requirements, pea breeding will rely on exploitable resources that can be found in the cultivated *Pisum sativum sativum* genepool as well as in the wild *P. sativum elatius*, *humile*, *abyssinicum* subspecies, and *P.fulvum* species [2-4]. Characterizing the structure of diversity in pea collections is useful both for conservation and exploitation of naturally occurring variability. Many diversity studies have been conducted in pea, so far mainly using Simple Sequence Repeat (SSR) markers [5-10] or polymorphisms of insertion sites of PDR1 *Ty1-copia* group retrotransposons (RBIP) [4,8,11]. More recently, next generation sequencing allowed rapid SNP discovery and genotyping array development in pea [12-15]. SNP markers have been widely used in a large range of living organisms both for genetic mapping and diversity assessment. They are abundant, well distributed in genomes and bi-allelic, all properties making them choice markers for population genetic approaches.

Crop genetic improvement has long relied on the phenotypic evaluation of related individuals and the calculation of their breeding value. This method has proved extremely successful but, as molecular marker technologies become more accessible, the potential of marker-assisted selection to significantly improve breeding of polygenic traits is broadening. The increasing availability of high-throughput genetic markers has prompted the development of new methodologies for identifying and targeting the molecular basis of complex phenotypes in breeding programs. In a seminal paper, Meuwissen et al. [16] proposed to use dense genotyping data from Single Nucleotide Polymorphisms (SNP) genotyping arrays as covariates in linear regression models for prediction of the genetic values for traits of interest. The method fits a predictive model in a “training population” of individuals for which both phenotypes and genotypes are known. Then, genomic estimated breeding values (GEBV) of test individuals are estimated using this model, based solely on genotypes. Individuals are finally selected based on their GEBV. The statistical apparatus of genomic selection is still in development and a whole range of methods have been devised [17,18]. As for genetic association studies, the question of the effect of genetic structure within the training population on the accuracy of the prediction is important. Genetic structure tends to create spurious signals of association in the detection of marker-phenotype associations due to extended linkage disequilibrium [19]. A proper description of diversity and population structure thus appears a significant prerequisite to such approaches. A variety of methods aimed at investigating genetic structure in natural populations as well as in collections of accessions are available [20-23].

In this paper, our objective was to test the ability of a set of newly developed SNP markers to describe the structure and to predict the phenotypes of a collection of 372 diverse pea accessions. We analysed the phenotypic and genotypic diversity of this collection including landraces and cultivars of garden, field or fodder peas as well as wild peas. Three types of markers -SNP, SSR, and RBIP markers- characterized by their contrasted mode of evolution, were used to provide a picture of the genetic diversity of this set of accessions. Then, different statistical methods were used to infer the structure of the collection and to predict some phenotypic traits from marker information. Despite the low density of markers used for prediction, promising results were obtained.

## Methods

### Plant material, genotyping and phenotyping

The 372 pea accessions used in this study are listed in Additional file 1 and at https://urgi.versailles.inra.fr/siregal/siregal/grc.do. This collection was gathered to represent a large sample of cultivated types as well as a large diversity including wild genotypes, world landraces and old cultivars. Passport information was available for part of the accessions, including the type of use (239 accessions classified into 92 garden, 7 mangetout, 13 preserve, 69 field or 58 fodder accessions), the type of population (302 accessions classified into 152 cultivar, 57 breeding lines, 52 landraces, 22 germplasm, 19 wild accessions), the type of sowing (242 accessions classified into 157 spring and 85 winter sown accessions). This collection was genotyped using the RBIP assay [4] and using SNP [12]. SSR genotyping was adapted from Jestin et al. [24]. Labelled PCR fragments were produced in one step in a total volume of 6.5 *μ*l with 5’ extensions in order to facilitate the labelling procedure at low cost (forward primer with the CACGACGTTGTAAAACGAC sequence extension) Genomic DNA (25 ng) was amplified with the following PCR mix: 2.345 *μ*l of dH2O, 2.3 mM of MgCl2 buffer, 1.35 mM of dNTPs, 0.04 U/ *μ*l Qiagen ®;Taq, 50 nM of forward primer with M13 tail, 500 nM of reverse primer, and 0.52 *μ*M of labeled M13 primer (6-FAM and NED, Applied Biosystems). The PCR program was 95° C for 5 min; 7 cycles of 95° C for 30 s, 62° C for 30 s (with a decrease of 1° C per cycle), 72° C for 30 s; 30 cycles of 95° C for 30 s, 55° C for 30 s, 72° C for 30 s; and a final extension at 72° C for 5 min. 2 *μ*l of the PCR product was then diluted (1/50) and pooled with 0.2 *μ*l LIZGS500 ladder and 9 *μ*l HI-Di Formamide (Applied Biosystems). Fragments were separated by capillary electrophoresis on ABI3130xl (Applied Biosystems) and data were analysed using GeneMapper 3.7 software.

**Figure 1:**
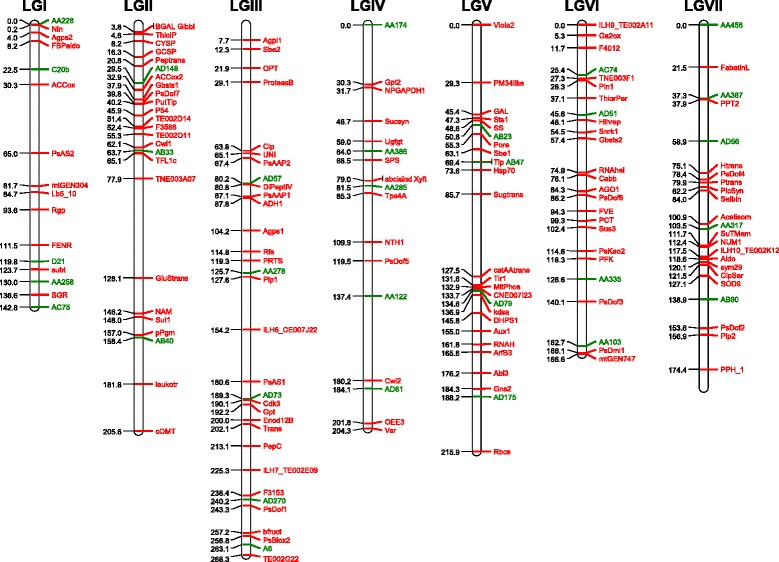
**Genetic positions of the markers used.** When their map positions were available, the markers used in the present study were placed on the pea genetic map according to [25]. SSR (Simple Sequence Repeat) markers are in green and SNP (Single Nucleotide Polymorphism) markers are in red.

Additional file 2 lists the 29 SSR, 31 RBIP and 351 SNP markers used in this study and Figure 1 displays the positions of the markers placed on the pea genetic map according to Bordat et al. [25]. On this map, 274 SNP corresponded to 129 mapped loci. All genotypes were tested in field experiments at Dijon-Epoisses (21110 Bretenière, France, 47.242° C North ans 5.114° C East) in 2003 and 2007. In 2003, 5 seeds per genotype were sown on a 1 m row, in a one-block design with controls, on the 27^th^ February 2003. Depending on plant germination and health, 1 (3 genotypes) to 5 (234 genotypes) plants per genotype were manually harvested at maturity. In 2007, 30 seeds were sown on a 2 m row in a two randomly replicated block trial on the 5^th^ March 2007, as described in [26]. Eight to ten central plants were manually harvested in each row, in order to minimize border effects. Plants were harvested according to their maturity. Several phenotypic traits were scored for each accession throughout the growing cycle. These include the date of beginning of flowering (expressed as a sum of daily temperatures above 0° C since sowing), the number and the weight of seeds per plant at the harvest. Thousand seed weight was calculated as a thousand time the ratio of the weight to the number of seeds.

### Statistical analysis

For all markers, the number of alleles, the polymorphic index content (PIC) and the minor allele frequency (MAF) were computed. For all pairs of accessions and for each type of markers, pairwise distances (or Rogers’ distances, RD [27]) were computed as the total number of polymorphic markers on the total number of markers scored. The mean number of polymorphic markers, the mean PIC and mean RD were calculated for each type of markers. The correlations among the three distance matrices obtained from the three types of markers were tested using Mantel’s test available on the package ade4 [28] of R software [29]. The heatmap of pairwise similarities associated with the hierarchical complete linkage clustering of accessions based on the similarity (1−*RD*) matrix (heatmap.2 function in gplots R package [30]) revealed genetic relationships among accessions of the collection. The structure of the collection was also analysed using Discriminant Analysis of Principal Components (DAPC) [23] and INSTRUCT [21]. DAPC relies on a transformation of the data by PCA thus ensuring that the variables of the transformed data are uncorrelated and their number is less than that of analysed individuals, followed by a discriminant analysis step. Since this method requires prior information about grouping of the individuals, a sequential k-means algorithm provided by the R package adegenet was run [31]. The optimal number of clusters to describe the data is determined from the Bayesian Information Criterion (BIC) plot as a function of the number of clusters. INSTRUCT uses a Bayesian clustering algorithm similar to the algorithm implemented in the widely used STRUCTURE program [20] but allows for partial inbreeding. Analyses were conducted allowing for admixture, for a number of groups K ranging from 1 to 7 with 5 MCMC chains per value of K, a burn-in period of 50,000 and a total of 100,000 iterations. The putative optimal K value was assessed by plotting the *ΔK* value, following Evanno et al. [32]. ANOVA were performed on the discriminant axes of the DAPC analysis to detect the effect of passport information on the structure (proc GLM, SAS Institute). The phenotypic values of the 2007 field trial were analysed by a two-way ANOVA with a genotype and a block effect, and the mean values of phenotypic variables were calculated for each accession (proc GLM, SAS Institute). Broad-sense heritabilities were calculated as 1−1/*F*, with F being the F value of the genotypic effect. Linkage disequilibrium *r*^2^ values were calculated among 129 linked loci corresponding to 274 SNP using EggLib [33] and plotted against the genetic distance among markers, according to the genetic map in [25].

### Genetic prediction

For the analyses of marker-phenotype association, 20 SNP markers were excluded from all the analyses due in particular to *MAF*≤2*%*, too many missing data, or heterogeneous genotyping, resulting in 331 SNP which were used for analyses. Phenotypes were available for 367 accessions. Other missing data were imputed using Probabilistic Principal Component Analysis available in pcaMethods package [34] of Bioconductor [35]. In the objective of predicting phenotypes from genotypic data, we compared the predictive performances of 6 statistical methods: LASSO (Least Absolute Shrinkage and Selection Operator) [36], PLS (Partial Least Squares) [37], SPLS (Sparse Partial Least Squares) [38], Bayes A, Bayes B [16] and GBLUP (Genomic Best Linear Unbiased Prediction) [39]. These methods were tested on TSW, BegFlo and NSeed phenotypes measured in 2003 and 2007. To avoid potential spurious associations caused by population structure, we used a new approach proposed by Johnson et al. [40] and based on an Empirical Bayes (EB) algorithm to correct this undesirable effect. This method was designed to adjust for batch effects in microarray datasets. This analysis was performed using the Combat function of the SVA package of R software [41]. Once this pre-processing step performed, the PLS, SPLS and LASSO methods were again used to predict the phenotypes.

#### LASSO (Least absolute shrinkage and selection operator)

LASSO [36] is a penalized linear regression model. It consists in minimizing the quadratic error under a constraint on the l1-norm of coefficients vector. It results in a sparse solution to the following optimization problem:

(1) $$ min \left\|y-\sum\limits_{j=1}^{p} x_{j} \beta_{j}\right\|^{2}_{2},  $$

(2) $$ \text{under the constraint} \left\|\beta\right\|_{1}=\sum\limits_{j=1}^{p}\left|\beta_{j}\right|\leq t,  $$

where *y* is the phenotypic trait of interest, *x*_*j*_ the genotype vector for the *j*^*th*^ SNP, *p* the total number of SNPs and *t* a tuning parameter.

The major interest of LASSO is that it allows a variable selection: the constraint induces nullity for some coefficients *β*_*j*_. The level of sparsity depends on the *t* parameter. The smaller the parameter, the less variables in the model. This enables to select the most relevant SNPs when predicting the phenotype. The optimal *t* value was chosen by 10-fold cross-validation as the one minimizing the Mean Square Error of Prediction. LASSO analyses were performed using the lars R-package [42].

#### PLS (Partial least squares) and SPLS (Sparse partial least squares)

Introduced by Wold in 1966 [37], the PLS regression is a statistical method which consists in regressing a variable *Y* on a set of *p* quantitative variables forming a *X* matrix. This method is particularly adapted in the case of high-dimensional data to avoid multi-colinearity problems. Its principle is based on the transformation of the initial variables space into a lower dimension space. To that end, new components *T* called latent variables are computed in an iterative way; these components are linear combinations of the initial variables and constrained to be orthogonal. They are constructed such as to maximize the covariance between *X* and *Y* and verify:

(3) $$ T=XW^{*}  $$

where *W*^∗^ is the loading matrix such as:

(4) $$ w^{*}_{h}=\text{argmax}cov(t_{h},Y)  $$

with $w_{h}^{\prime }w_{h}=1$ and $t_{h}^{\prime }t_{j}=0$ for *j*<*h*.

The loadings $w_{h}^{*}$ expresses the importance of each of the variables in the construction of the new components *t*_*h*_. The first components are sufficient to explain most of the data covariance.

The SPLS regression [38] is a method derived from PLS which simultaneously offers a variable selection. Variables are selected by introducing a LASSO penalization on the loadings. The number of variables to be included in each latent variable is chosen empirically: 50 SNPs were thereby selected on each component to facilitate the biological interpretation. We used the mixOmics package [43] of R software to perform both PLS and SPLS analyses.

#### GBLUP (Genomic best linear unbiased prediction)

VanRaden [39] suggested Genomic BLUP method where SNP markers are normally distributed with the same effects for all markers. The model considered is:

(5) $$ y=\mu+Zg+e  $$

where *y* is the vector of phenotype, *μ* the overall mean, *Z* the incidence matrix of markers effects, *g* the vector of SNP effects where $g\sim \mathcal {N}\left (0,G{\sigma ^{2}_{g}}\right)$. *e* is the vector of residual errors where $e\sim \mathcal {N}\left (0,{\sigma ^{2}_{e}}\right)$. *G* is the matrix of genomic relationship based on SNP and ${\sigma ^{2}_{g}}$ is the additive genetic variance. These analyses were performed using the rrBLUP R-package [44].

#### Bayesian methods: Bayes A and Bayes B

For some characters, only a few genes have large effects. To take into consideration this reality, Meuwissen et al. [16] proposed two Bayesian methods which enable different prior distributions of markers’ effects. They are based on the following model:

(6) $$ y=Xu+e  $$

where *y* is the phenotypic trait of interest, *X* the matrix of SNP markers, $u\sim \mathcal {N}\left (0,{\sigma ^{2}_{u}}\right)$ the vector of random SNP effects and $e\sim \mathcal {N}\left (0,{\sigma ^{2}_{e}}\right)$ is the vector of residual errors. The first method, Bayes A supposes that each SNP has a proper effect; this effect differs from one SNP to the other and the variance of each SNP is written as:

(7) $$ {\sigma^{2}_{u}}\sim \chi^{-2}(\nu,S)  $$

where *ν* is the number of degrees of freedom and *S* is the scale parameter.

On the other hand, Bayes B assumes that a proportion *π* (often important) of SNP has a null variance. The prior distribution of the variances is written as follows:

(8) $$ \left\{ \begin{array}{l l} {\sigma^{2}_{u}}=0 & \text{with probability \(\pi\)}\\ {\sigma^{2}_{u}}\sim \chi^{-2}(\nu,S) &\text{with probability \((1-\pi)\)} \end{array} \right.  $$

For *π*, a probability *π*=0.95 was empirically chosen.

Bayesian analyses were performed using the BGLR R-package [45] in which algorithms are based on a Gibbs Sampler.

#### Performance assessment of the methods

The ability of each model (using the statistical methods) to correctly predict the phenotype of test genotypes was evaluated using different parameters. To that end, the dataset was split into a training and a test set composed respectively by 2/3 and 1/3 of the data. This ratio is commonly chosen in such situation. The training set is large enough to provide reliable estimation of the model and the test set is large enough to give an accurate assessment of the predictive ability of the model. This process was repeated 500 times; each time, the test and training sets were sampled at random (without replacement). For each iteration, models were created on the training set, applied to the test set and evaluated by computing mean squared error of prediction (MSEP) with:

(9) $$ MSEP=\frac{1}{n_{test}}\sum\limits^{n_{test}}_{j=1}\left(y_{j}-\hat{y}_{j}\right)^{2},  $$

where *n*_*test*_ is the total number of samples in test set, *y*_*j*_ is the observed phenotype value for the *j*^*th*^ test object and $\hat {y}_{j}$ the value predicted on test set by the model creating with training set.

Moreover, the coefficient of determination *R*^2^ was calculated on training set as

(10) $$ R^{2}=1-\frac{RSS}{{TSS}_{train}}=1-\frac{\displaystyle{\sum\limits^{n_{train}}_{i=1}{\left(y_{i}-\hat{y}_{i}\right)^{2}}}} {\sum\limits^{n_{train}}_{i=1}{\left(y_{i}-\bar{y}_{train}\right)^{2}}}  $$

where *RSS* means the residuals sum of squares and *TSS*_*train*_ the total sum of squares. *n*_*train*_ is the total number of samples in the training set, *y*_*i*_ is the phenotype value for sample *i*, $\bar {y}_{\textit {train}}$ the mean of the observed trait of interest values and $\hat {y}_{i}$ the prediction by the training model on the *i*^*th*^ sample. *R*^2^ represents the proportion of variance explained by the model and is used to evaluate the goodness of fit of the model to data.

*Q*^2^ is analogous to *R*^2^ but was used to evaluate the prediction quality of the model :

(11) $$ Q^{2}=1-\frac{PRESS}{{TSS}_{test}}=1-\frac{{\sum\limits^{n_{test}}_{i=1}{\left(y_{i}-\hat{y}_{i}\right)^{2}}}} {\sum\limits^{n_{test}}_{i=1}{\left(y_{i}-\bar{y}_{test}\right)^{2}}}  $$

where *PRESS* is the predictive sum of squares and *TSS*_*test*_ the total sum of squares of the test set. $\bar {y}_{\textit {test}}$ indicates the mean of the test set. Unlike *R*^2^, *Q*^2^ can be negative when the model predictions are poor.

Finally, MSEP, *R*^2^ and *Q*^2^ were averaged over the 500 repetitions.

## Results

### Genetic diversity of a collection of 372 pea accessions

**Figure 2:**
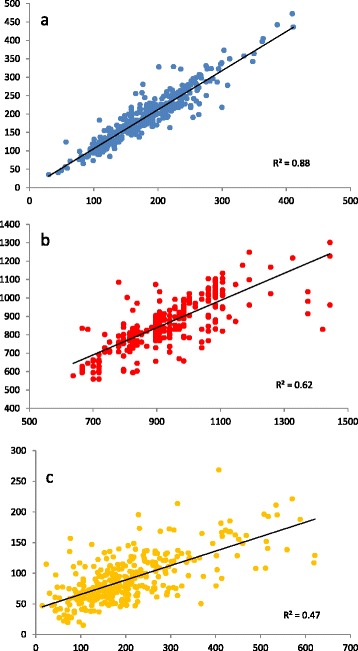
**Correlations of phenotypes between the 2003 and 2007 field trials.** **(a)** Thousand seed weight (g), **(b)** Sum of temperatures from sowing to beginning of flowering (degree.days) and **(c)** Seed Number per plant.

The broad cultivation range of pea was associated with a wide genetic variation both at the phenotypic and molecular levels. The levels of polymorphism revealed by the three types of markers used in this study were contrasted. It was rather low for RBIP markers, medium for SNP and high for SSR (Table 1 and Additional file 2). The proportion of monomorphic markers was almost zero for SNP and SSR probably because most of SSR and SNP markers were selected based on their polymorphism among a set of 5-8 genotypes [12,46]. It reached 13% for RBIP even if they had been selected for their informativeness [11]. Mean Polymorphic Information Content (PIC) and pairwise distances (RD) were higher for SSR (0.8), intermediate for SNP (0.33 and 0.38 resp.), and lower for RBIP (0.24 and 0.17 resp.). All SSR, 11% of RBIP and only 2.6% of SNP markers displayed a Minor Allele Frequency (MAF) below 1%. This may be related to the high number of alleles detected for the SSR markers (Additional file 3) whereas SNP and RBIP markers are by construction bi-allelic. The histogram of MAF frequency showed different distributions for SNP and RBIP markers (Additional file 4): while the proportion of RBIP markers rapidly decreased from low to high MAF values, the distribution of MAF values was even among SNP markers. For the three types of markers, the proportion of heterogeneous accessions (representing either heterozygote plants or more probably a mix of different homozygote plants) was low, ca. 1% (Table 1), as expected in a predominantly selfing species. As for molecular diversity, a wide and significant genetic variation was observed for the phenotypic traits analysed (Table 2): the time of Beginning of flowering (BegFlo) spanned between 637 to 1443 degree.days in 2003 and 540 to 1301 degree.days in 2007, the number of seeds per plant (NSeed) ranged respectively from 14.5 and 621 and 11.4 and 375, and the thousand seed weight from 29.9 to 410 and 35.6 to 472 g (Figure 2 and Table 2). Broad-sense heritabilities computed in the 2007 field trial were high (NSeeds) to very high (TSW and BegFlo). Consistently, correlations between 2003 and 2007 data were very high for TSW and BegFlo and moderate for NSeed (Figure 2) suggesting higher Genotype by Environment (GxE) interactions for this latter trait.

**Table 1:** **Summary statistics calculated for SSR (Simple Sequence Repeat), SNP (Single Nucleotide Polymorphism) and RBIP (Retrotransposon-based insertion Polymorphism) markers**

	**SSR**	**SNP**	**RBIP**
# Monomorphic	0	2	4
% Monomorphic	0.00	0.01	0.13
# Polymorphic	29	351	27
Mean RD	0.80	0.33	0.17
% Heterogeneous accession	1.4	0.83	1.26
% MAF <0.01	100	2.6	11
Mean PIC	0.80	0.38	0.24
Min PIC	0.46	0.01	0.01
Max PIC	0.97	0.99	0.95

RD: Rogers distance, MAF: minimum allele frequency, PIC: polymorphism information content.

**Table 2:** **Summary statistics on phenotypes**

**Variable**	**Mean**	**Min**	**Max**	**Std**	**F genotype**	**F block**	**P genotype**	**P block**	${h^{2}_{\textit {sl}}}$
**2007**									
BegFlo	858.45	540.00	1301.00	135.85	157.95	34.17	***	***	0.99
Nseed	89.08	11.44	375.18	42.00	3.47	0.007	***	ns	0.71
TSW	193.45	35.56	472.50	72.69	46.54	11.41	***	***	0.98
**2003**									
BegFlo	931.57	637.40	1443.90	220.66	na	na	na	na	na
Nseed	205.81	14.50	621.00	114.66	na	na	na	na	na
TSW	181.03	29.91	410.85	67.19	na	na	na	na	na

Mean, Minimum, Maximum and Standard deviation for beginning of flowering in degree.days (BegFlo), the number of seed per plant (Nseed), and thousand seed weight (TSW) in the 2003, 2007 field trials. Fisher’s test values and probability of the two-way ANOVA with genotype and block effects, and broad sense heritabilities were calculated for the 2007 field trial only.

### Structure of the population

The different marker types did not reveal the structure of the collection at the same granularity; while 6% of pairwise distances revealed by SSR reached 1 (100% of SSR markers polymorphic among the two accessions) and no pairwise distance was null, 2.5% of pairwise distances revealed by RBIP markers were null (no polymorphic RBIP among the two accessions) and no distance reached 1. Mantel’s correlation between SNP and SSR was 0.6 (*P*<10^−5^) whereas correlations between RBIP distance and both SNP and SSR distances were lower (0.33 and 0.35 respectively, *P*<10^−5^). Mean distances between accessions of the same or of different use types were computed, in order to see if pairs of accessions taken within the same use type were more closely related than pairs of accessions taken from different use types. For SNP markers, intra-use type pairwise distances were slightly lower, on average, than inter-use type distances (Table 3). Distances between fodder accessions and other types (field and garden) were higher on average, than distance between field and garden accessions. For SSR markers, intra-use type distances were lower than inter-use type distances for garden and field accessions whereas distances intra-fodder use type were similar to distances between fodder and other use type accessions (Table 3).

**Table 3:** **Average pairwise distances between accessions of the same (italics) or of different groups of use types: Average distances were computed among and within each of the 3 groups of use, field, fodder or garden peas, for each of the 3 types of markers used**

	**USE**	**Field**	**Fodder**	**Garden**
**a. SNP**	Field	*0.30*	0.35	0.32
	Fodder	0.35	*0.33*	0.36
	Garden	0.32	0.36	*0.28*
**b. SSR**	Field	*0.70*	0.82	0.75
	Fodder	0.82	*0.81*	0.82
	Garden	0.75	0.82	*0.69*
**c. RBIP**	Field	*0.12*	0.18	0.13
	Fodder	0.18	*0.20*	0.19
	Garden	0.13	0.19	*0.14*

**Figure 3:**
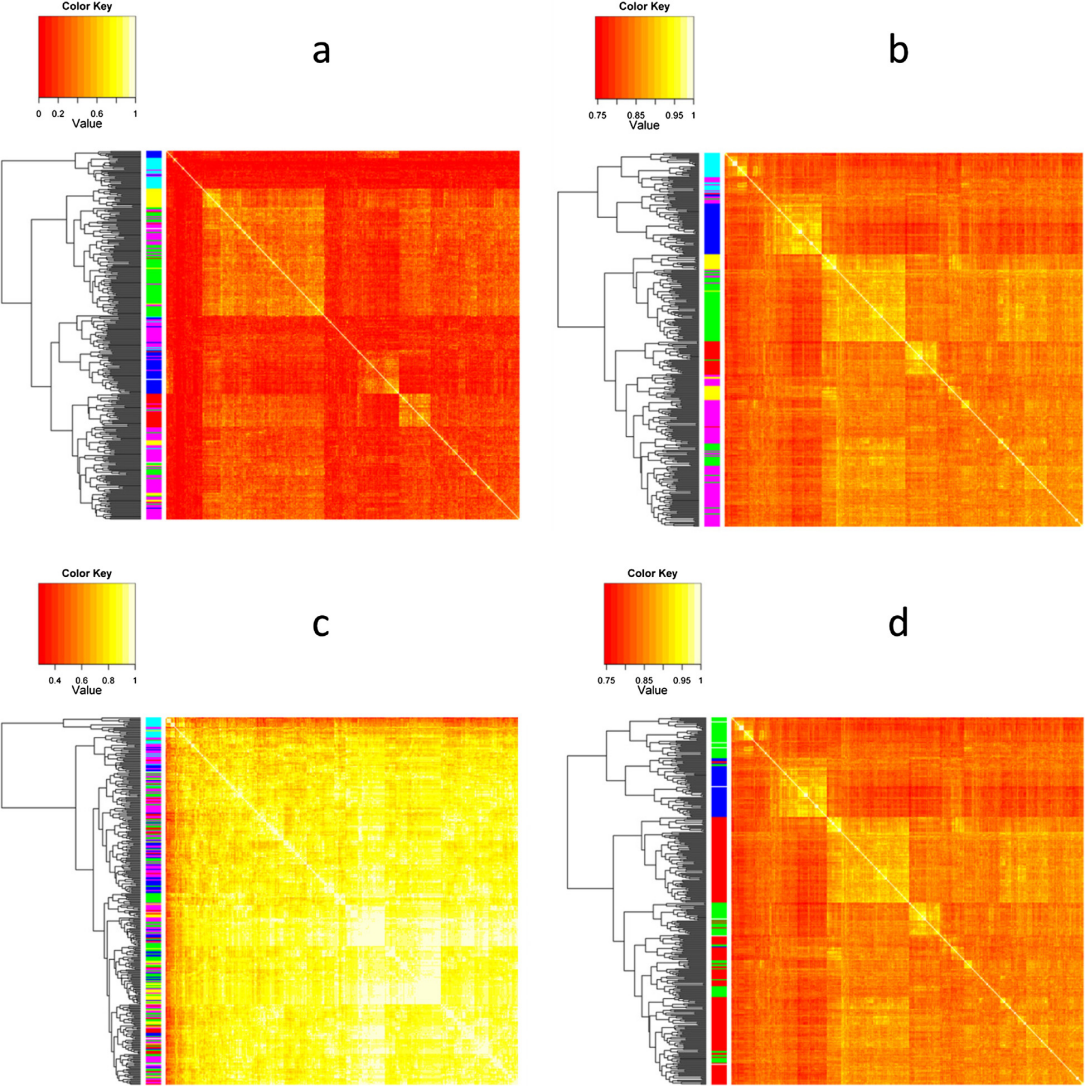
**Heatmaps of genetic similarities calculated for the different marker types.** Dendograms represent complete linkage clustering of the 372 accessions according to **(a)** SSR markers, **(b and d)** SNP markers, **(c)** RBIP markers. The 6 DAPC (Discriminant Analysis of Principal Component) groups **(a, b, c)** and the 3 INSTRUCT group **(d)** are represented by the left colored banner.

**Figure 4:**
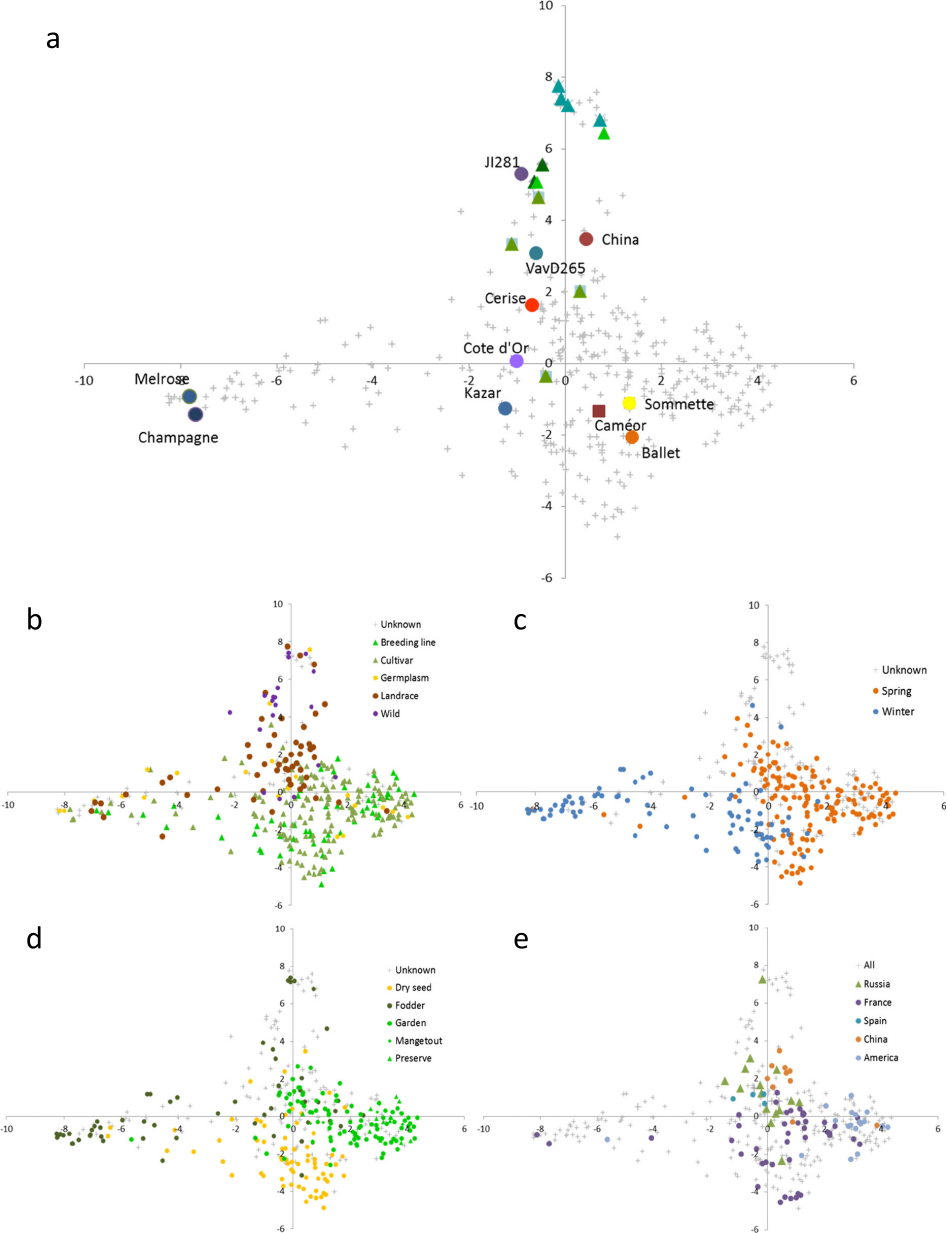
**First plan of the DAPC (Discriminant Analysis of Principal Component) analysis revealed genetic diversity of the collection.** The analysis was performed on SSR and SNP data. **(a)** Dots highlight parents of recombinant inbred line populations, and triangles highlight *P.sativum elatius* (violet), *P.sativum abyssinicum* (blue), *P.sativum humile* (green) and *P. fulvum* accessions (orange). Accessions are also represented according to their population type **(b)**, sowing type **(c)**, use type **(d)** and geographical origins **(e)**.

For RBIP markers, intra and inter distances for field and garden accessions were similar, and intra and inter distance for fodder accessions were higher (Table 3). The different markers also revealed different levels of diversity among accessions according to population type (Table 4). For the three types of markers, pairwise distances among cultivars and breeding lines were lower, while distances among wild accessions and between wild accessions and other types were higher, on average, than other comparisons. RBIP distances between wild and other accessions were, on average, twice as large as distances among cultivars, breeding lines, landraces and germplasm, while this ratio was only ca. 1.3 for SNP and SSR. The heatmap and hierarchical clustering of genetic similarities among the collection showed that SNP markers (7 groups differentiated) more clearly distinguished structure than SSR for which most distances were high (4 groups) or RBIP for which most distances were low (2 groups; Figure 3). The structure of the collection was further investigated using two other approaches: the DAPC approach divided the collection into six groups (Additional file 1, Figure 3): group 1 included 34 accessions, mainly spring field pea cultivars; group 2 included 32 accessions, mainly winter field pea cultivars, group 3 included 89 accessions, mainly spring garden pea cultivars and breeding lines, group 4 included 32 accessions, mainly wild peas and landraces, group 5 included 52 accessions, mainly winter fodder peas, and group 6 included 130 accessions, mainly spring cultivars and landraces. The first discriminant plan of the DAPC analysis showed significant genetic diversity among specific accessions, including parents of the current pea consensus map [25] and wild accessions from the *Pisum fulvum*, *P. sativum elatius*, and *P. sativum abyssinicum* subspecies. It also showed that accessions generally clustered according to their use, population, sowing types as well as geographical origins (Figure 4). This was confirmed by the analysis of variance on the coordinates of the accessions on the 5 first axes of the DAPC analysis, which showed significant effects of the passport data on the different axes (Additional file 5). The third approach to characterize genetic structure was INSTRUCT. INSTRUCT divided accessions into three groups (Additional file 6): group 1 was composed of 211 accessions, mainly spring garden pea cultivars. Group 2 gathered mainly spring field pea cultivars and landraces; group 3 included mainly winter fodder peas. More admixed individuals were found with INSTRUCT than with DAPC. Considering *K*=3, 29.2% of the individuals were admixed at a 20% threshold using INSTRUCT vs 13.4% admixed individuals using DAPC. As for passport classes, we calculated mean pairwise distances among accessions from the same or from different INSTRUCT or DAPC groups (Additional file 7). On average, distances between accessions from the same DAPC or INSTRUCT group were smaller than distances among accessions from different groups, except for DAPC group 4 and 6 and INSTRUCT group 2, that showed similar intra-group and inter-group mean distances for SSR and RBIP markers. The hierarchical clustering obtained from pairwise similarities (Figure 3), where accessions were colour coded according either to the three INSTRUCT groups or to the six DAPC groups, showed that the different methods reveal somewhat different clustering, SNP hierarchical clustering being the most congruent with INSTRUCT or DAPC groupings.

### Prediction results

**Figure 5:**
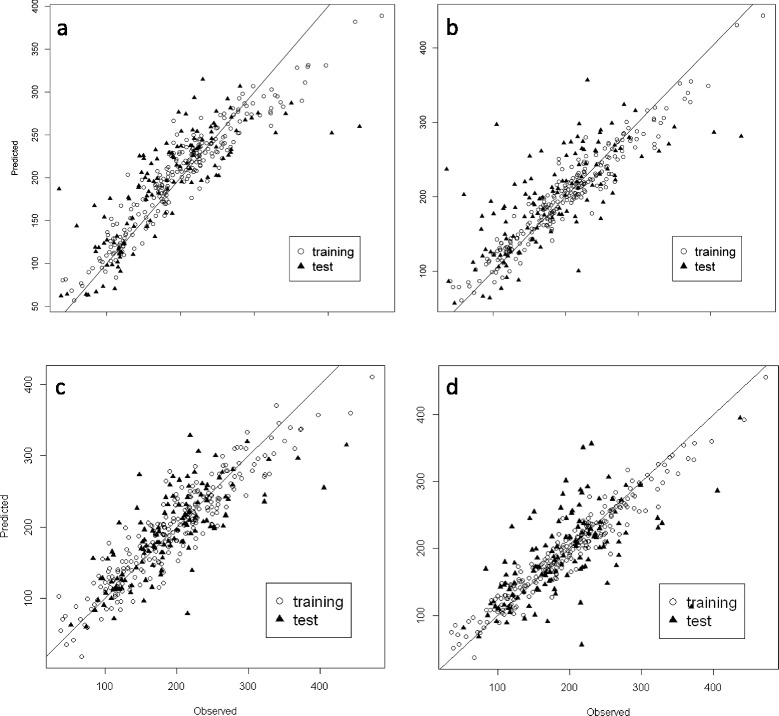
**Plots of observed vs predicted Thousand Seed Weight (TSW) values in the 2007 field experiment.** Predicted values after **(a)** the Genomic Best Linear Unbiased Prediction (GBLUP) method, **(b)** the Least Absolute Shrinkage and Selection Operator (LASSO) method, **(c)** the Partial Least Squares (PLS) method taking into account the INSTRUCT structure; **(d)** the LASSO method taking into account the DAPC structure.

**Figure 6:**
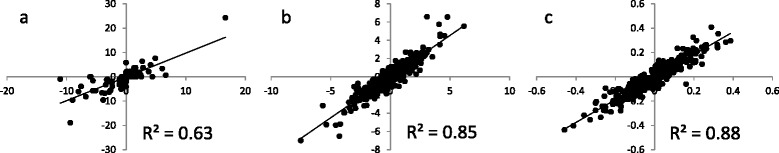
**Correlation of SNP Prediction coefficients for TSW in 2003 and 2007 according to different statistical methods.** **(a)** Least Absolute Shrinkage and Selection Operator (LASSO) method coefficients **(b)** Genomic Best Linear Unbiased Prediction (GBLUP) method coefficients and **(c)** Partial Least Squares (PLS) method coefficients.

**Figure 7:**
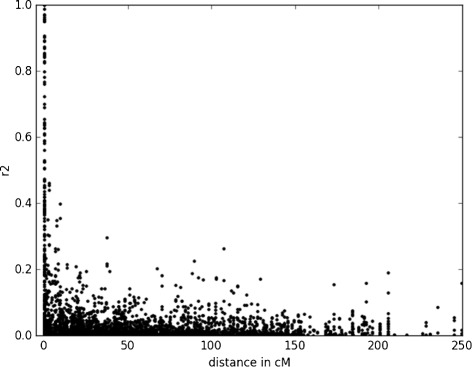
**Linkage disequilibrium (** ***r*** ^**2**^ **) as a function of genetic distance among linked markers, in cM.**

Table 5 presents the predictive performances for the PLS, SPLS, LASSO, Bayes A, Bayes B and GBLUP models for three phenotypic traits measured in 2003 and 2007. *Q*^2^ expresses the fit of predicted and actual phenotypes in the test set and *R*^2^ the fit in the training set. The highest *Q*^2^ values were obtained using GBLUP for 4 traits and PLS for 3 traits. TSW was better predicted than BegFlo and NSeed (Figure 5). Interestingly, the SNP prediction coefficients obtained for TSW in 2003 and 2007 were significantly correlated (from *R*^2^=0.62 with LASSO to *R*^2^=0.88 with PLS, Figure 6). Additional file 8 shows the most predictive SNP for TSW with the six methods for both 2003 and 2007 data. Some SNP were consistently found among the most predictive variables of the 12 models (agps2_SNP1,3; Hsp70_SNP1; RNAH_SNP4; At2g44950_SNP1, Bfruct_SNP1-4-7; ACCox_SNP3; CE007E18_SNP1; CE007H08_SNP1). In order to assess their possible contribution due to linkage with causal genes, we calculated the linkage disequilibrium (LD) among linked SNP markers. LD was high at low distance (notably among different SNP in the same gene) and moderate to low at distances larger than a few centiMorgans (Figure 7). In order to test for the effect of structure as revealed by DAPC and INSTRUCT on the quality of the prediction, the ComBat correction was applied to subtract this effect. Additional file 9 shows that this method correctly adjusted for population structure. The prediction of phenotypes was done using data adjusted from Combat to avoid spurious associations between SNP and phenotypes. Table 5 presents the predictive performances for the PLS, SPLS and LASSO after adjusting the datasets for structure. The correction by INSTRUCT did not significantly changed *Q*^2^ values of the PLS and SPLS prediction of TSW. But in all other cases, the correction for structure led to decreased *Q*^2^ values indicating a lower accuracy of prediction. The number of SNP contributing to the prediction decreased after correcting for structure (Additional file 10). It decreased more after DAPC correction than after INSTRUCT correction. The PLS method retained the largest number of predictive variables and the LASSO method retained less variables.

**Table 4:** **Average pairwise distances between accessions from the same (italics) or from different groups of population types: Average distances were computed among and within each of the 5 population types: breeding lines, cultivars, germplasm, landraces, wild genotypes, for each of the 3 types of markers used**

	**POP**	**Breeding**	**Cultivar**	**Germplasm**	**Landrace**	**Wild**
**a. SNP**	breeding	*0.30*	0.31	0.33	0.34	0.38
	cultivar	0.31	*0.31*	0.34	0.34	0.37
	germplasm	0.33	0.34	*0.34*	0.35	0.37
	landrace	0.34	0.34	0.35	*0.34*	0.36
	wild	0.38	0.37	0.37	0.36	*0.34*
**b. SSR**	breeding	*0.70*	0.73	0.80	0.82	0.89
	cultivar	0.73	*0.73*	0.80	0.82	0.89
	germplasm	0.80	0.80	*0.81*	0.85	0.90
	landrace	0.82	0.82	0.85	*0.84*	0.90
	wild	0.89	0.89	0.90	0.90	*0.93*
**c. RBIP**	breeding	*0.13*	0.13	0.15	0.17	0.33
	cultivar	0.13	*0.13*	0.16	0.18	0.34
	germplasm	0.15	0.16	*0.18*	0.19	0.33
	landrace	0.18	0.18	0.19	*0.20*	0.33
	wild	0.33	0.34	0.33	0.33	*0.36*

**Table 5:** **Performances of the Least Absolute Shrinkage and Selection Operator (LASSO), Partial Least Square (PLS) and Sparse Partial Least Squares (SPLS), Bayes A, Bayes B and Genomic Best Linear Unbiased Prediction (GBLUP) methods to predict phenotypes measure in field trials in 2003 and 2007, with or without taking into account the genetic structures revealed by DAPC and INSTRUCT**

	**Thousand seed weight**		**Beginning of flowering (** $\sum T{}^{\circ}C$ **)**		**Seed number**	
	**2003**	**2007**	**2003**	**2007**	**2003**	**2007**
**MSEP**						
LASSO	1931 (360)	2185 (398)	16074 (2469)	10953 (1511)	7722 (1035)	1047 (180)
LASSO-DAPC	2334 (404)	2761 (458)	17893 (3018)	13080 (1814)	9700 (1598)	1342 (206)
LASSO-INSTRUCT	1882 (364)	2455 (511)	18019 (2623)	13013 (1805)	9566 (1629)	1310 (213)
PLS	1737 (283)	2011 (312)	14733 (2196)	9883 (1247)	7978 (1019)	1003 (163)
PLS-DAPC	2058 (296)	2414 (333)	16292 (2371)	11882 (1317)	10531 (1481)	1234 (215)
PLS-INSTRUCT	1790 (249)	2040 (300)	17357 (2346)	11947 (1430)	9786 (1410)	1226 (211)
SPLS	1947 (286)	2215 (332)	16064 (2160)	11546 (1441)	7820 (985)	1079 (188)
SPLS-DAPC	2355 (321)	2696 (399)	18929 (2493)	13115 (1725)	10476 (2216)	1337 (214)
SPLS-INSTRUCT	1906 (285)	2204 (316)	18250 (2653)	12644 (1537)	9947 (1887)	1336 (215)
Bayes A	1822 (329)	2084 (344)	14823 (2517)	10099 (1431)	7631 (1020)	1304 (421)
Bayes B	1814 (327)	2051 (350)	14722 (2471)	10206 (1474)	7660 (1002)	1328 (408)
GBLUP	1759 (288)	2017 (342)	14237 (2287)	9858 (1301)	7593 (988)	1296 (409)
***R*** ^**2**^						
LASSO	0.78 (0.06)	0.81 (0.04)	0.56 (0.11)	0.71 (0.09)	0.65 (0.07)	0.62 (0.11)
LASSO-DAPC	0.79 (0.08)	0.88 (0.04)	0.55 (0.15)	0.70 (0.11)	0.59 (0.11)	0.52 (0.16)
LASSO-INSTRUCT	0.88 (0.06)	0.91 (0.06)	0.49 (0.15)	0.70 (0.11)	0.59 (0.12)	0.52 (0.16)
PLS	0.77 (0.01)	0.79 (0.01)	0.56 (0.02)	0.76 (0.02)	0.70 (0.02)	0.69 (0.02)
PLS-DAPC	0.79 (0.01)	0.80 (0.01)	0.50 (0.02)	0.56 (0.02)	0.68 (0.02)	0.51 (0.03)
PLS-INSTRUCT	0.83 (0.01)	0.84 (0.01)	0.47 (0.03)	0.69 (0.02)	0.61 (0.02)	0.62 (0.02)
SPLS	0.74 (0.02)	0.77 (0.02)	0.59 (0.03)	0.79 (0.02)	0.67 (0.02)	0.55 (0.03)
SPLS-DAPC	0.71 (0.02)	0.73 (0.02)	0.40 (0.03)	0.73 (0.02)	0.46 (0.03)	0.41 (0.03)
SPLS-INSTRUCT	0.88 (0.01)	0.85 (0.01)	0.41 (0.03)	0.61 (0.02)	0.57 (0.03)	0.55 (0.03)
Bayes A	0.84 (0.03)	0.87 (0.02)	0.69 (0.06)	0.81 (0.03)	0.70 (0.04)	0.62 (0.11)
Bayes B	0.83 (0.03)	0.87 (0.02)	0.68 (0.06)	0.81 (0.04)	0.70 (0.04)	0.62 (0.10)
GBLUP	0.85 (0.02)	0.88 (0.02)	0.69 (0.05)	0.82 (0.03)	0.70 (0.04)	0.62 (0.10)
***Q*** ^**2**^						
LASSO	0.50 (0.07)	0.58 (0.06)	0.20 (0.07)	0.40 (0.71)	0.38 (0.08)	0.32 (0.07)
LASSO-DAPC	0.40 (0.10)	0.47 (0.09)	0.11 (0.12)	0.30 (0.09)	0.23 (0.10)	0.11 (0.09)
LASSO-INSTRUCT	0.52 (0.09)	0.53 (0.10)	0.11 (0.08)	0.29 (0.09)	0.25 (0.10)	0.14 (0.10)
PLS	**0.55 (0.06)**	0.61 (0.05)	0.27 (0.07)	**0.46 (0.06)**	0.35 (0.09)	**0.34 (0.08)**
PLS-DAPC	0.47 (0.07)	0.54 (0.06)	0.19 (0.08)	0.35 (0.07)	0.17 (0.11)	0.19 (0.11)
PLS-INSTRUCT	0.54 (0.06)	0.61 (0.05)	0.13 (0.09)	0.35 (0.08)	0.22 (0.11)	0.19 (0.11)
SPLS	0.49 (0.07)	0.58 (0.05)	0.20 (0.09)	0.36 (0.08)	0.37 (0.09)	0.29 (0.08)
SPLS-DAPC	0.39 (0.09)	0.48 (0.08)	0.06 (0.11)	0.28 (0.10)	0.17 (0.18)	0.12 (0.10)
SPLS-INSTRUCT	0.51 (0.08)	0.58 (0.06)	0.10 (0.09)	0.30 (0.09)	0.21 (0.15)	0.12 (0.12)
Bayes A	0.53 (0.06)	0.61 (0.05)	0.26 (0.07)	0.45 (0.07)	**0.39 (0.07)**	0.27 (0.11)
Bayes B	0.53 (0.06)	0.61 (0.05)	0.27 (0.07)	0.44 (0.07)	0.38 (0.08)	0.26 (0.10)
GBLUP	0.54 (0.06)	**0.62 (0.05)**	**0.29 (0.070)**	**0.46 (0.07)**	**0.39 (0.08)**	0.28 (0.10)

The best predictions are highlighted in bold. Mean square error of prediction (MSEP) indicates expected squared Euclidian distance between predicted and observed phenotypes, *R* ^2^ expressed the proportion of variance explained by the model and *Q* ^2^ evaluates the prediction quality of the model. Standard deviations are in parentheses.

## Discussion

### The different marker types reveal genotype divergence at different timescales

In this study, the levels of polymorphism exhibited by SSR, RBIP, and newly developed SNP markers [12] were compared for the first time in a broad pea germplasm collection. This comparison showed that these markers largely differed in their level of polymorphism and suggested the fastest rate of evolution for SSR, intermediate rate for SNP and slower rate for RBIP markers. While RBIP tend to shrink recently evolved diversity with one group corresponding to cultivated germplasm and two groups corresponding to wild genotypes, SSR seemed to saturate longer-term evolution and did not differentiate population types as clearly as SNP (Figure 3 and Table 4). RBIP have proved well suited for the study of *Pisum* species, subspecies, and primitive accessions relationships. Jing et al. [11] inferred a model for *Pisum* domestication based on RBIP patterns of variation among a large collection of wild and primitive *Pisum* accessions present in the JIC collection. Jing et al. [4] characterized European germplasm collections using RBIP markers and identified original *P. abyssinicum* and *P. elatius* accessions from the Polish collection as well as probably primitive pea accessions in the Dutch collection as compared to other European collections. Nevertheless, our results contrasted with the hypothesis that the mutation rate of RBIP markers was 100 higher than that of SNP. RBIP markers mutation rate was estimated around 5.10^−7^ per generation in pea [4,47,48], which was hypothesized a slightly higher rate of evolution as compared to SNP (ca 10^−8^−10^−9^ by Jing et al. [49]). However, this estimation was based on a limited number of genes and genotypes. SSR on the other hand, are most suited for studying recently evolved genetic variation. SSR are known to evolve at fast rate. This is precisely why they were widely used in forensic genetics in human [50]. Cieslarová et al. [51] estimated the mutation rate of some SSR markers ca 5.10^−3^ per generation in pea. Smýkal et al. [52] showed that SSR could differentiate accessions issued from the same genotype after long term storage in genebanks. In the present study, SSR revealed a large number of alleles (Additional file 3) in the collection and efficiently differentiated recently evolved cultivars. Finally, SNP appeared the most efficient to render the whole range of genetic distance among the accessions of our collection. Large sets of SNP that are now available in pea should be useful both for germplasm issued from longer term evolution and for recently evolved cultivars. Furthermore, low MAF values (below 1%) that were observed for all SSR, probably due to their highly multi-allelic nature and high mutation rate, and for 11% of RBIP, probably due to their low mutation rate in recently evolved germplasm, make these types of markers less suitable than SNP for association studies.

### Genetic structure of the pea germplasm

Our pea collection is a subset of the INRA collection and includes wild germplasm from the core16 of the JIC collection as well as landraces and cultivars from China, Russia, USA and Spain. This collection of 372 accessions was gathered in order to represent a wide range of phenotypic and passport data diversity. We confirmed (i) that the pea germplasm is structured according to major passport classes as defined by the use type, the population type, and the sowing type and (ii) that the range of genetic variability present in the *Pisum sativum* germplasm is high, as already pointed out by other authors [4,5,8,9,53]. However, this passport information may be misleading in some cases: the fodder and winter-sowing types are ancestral characteristics present in wild germplasm but can correspond in the collection to contrasted levels of winter tolerance and to different growth habits. In our classification, the fodder type may correspond either to a slender highly branched bushy type (former *P. arvense*) or to a tall vining intermediately branched type. In this study, we showed that SNP markers significantly help refining the genetic relationship among the cultivated genepool. Interestingly, even if the genetic diversity present in wild and primitive *Pisum sativum* accessions is larger than in the cultivar and breeding germplasm (Table 4), a substantial variability is retained in the cultivated pool. Similarly, Vershinin et al. [47] showed that most alleles from the wild genepool were still present in the cultivated genepool but in a lower number of combinations. We hypothesize that the genetic diversity of *Pisum sativum* was probably maintained thanks to the diversity of uses and to the contrasted environmental conditions and crop management of the different producing areas. This suggests that there is still a useful reservoir of diversity present in the worldwide cultivated genepool. Several authors similarly mentioned the continuum of variation among the *Pisum* genus. The number of groups that best represent the diversity is thus not easy to define. Jing et al. [11] defined, among the 4538 accessions of main European collections, 3 main groups using 27 RBIP markers, one group with *Pisum* subspecies, and two groups of *Pisum sativum sativum*. Zong et al. [9] using 21 SSR markers clustered 2120 accessions of the Chinese and Australian collections into 6 to 8 groups; Smýkal et al. [54] defined respectively 8, 14, and 9 groups within the 2120 accessions of the ATFC collection, the 3029 accessions of the JIC collection, and the 1283 accessions of the Czech collection. Structure description is not unequivocal and group ascertainment is potentially prone to misassignments, probably because of (i) the difficulty to summarize all the information contained in large datasets (many variables and/or accessions) and (ii) marker information that may not be complete enough. Most studies conducted so far were based on rather limited number of markers probably introducing sampling biases. Baranger et al. [5] mentioned that increasing the number of markers by pooling different types of markers was efficient in getting a clearer picture of genetic relationships. The markers used in the present study (Figure 1) represent the largest set used so far to characterize pea germplasm. Different methods describe genetic relationships among germplasm accessions. The methods proposed by STRUCTURE and INSTRUCT are extremely popular but rely on an explicit population genetics model which might make them sensitive to large departures from the postulated model. Furthermore, when the dataset is large, the computational burden incurred can be substantial. DAPC, which makes no assumption on population genetics parameters has been increasingly applied to diversity studies and performed well especially in the case of large datasets [23,55]. In the present paper, the distance-based, INSTRUCT, and DAPC methods proved complementary. The congruence of hierarchical clustering based on SNP distances with INSTRUCT and DAPC is well visualized on Figure 3.

### Genomic prediction of important traits

In this study, several approaches were tested for the first time to predict phenotypes from genotypes in pea. Thousand seed weight (TSW), Beginning of Flowering (BegFlo) and Number of Seeds per plant (NSeed) measured in two field trials were predicted in our collection from the genotypes at 331 SNP using six different methods (Partial Least Squares -PLS, Sparse Partial Least Squares -SPLS, Least Absolute Shrinkage and Selection Operator -LASSO, Bayes A, Bayes B and Genomic Linear Unbiased Prediction –GBLUP). Prediction results were promising: *Q*^2^ values ranged from 0.29 and 0.46 for BegFlo, 0.39 and 0.34 for NSeed to 0.55 and 0.62 for TSW, in 2003 and 2007 respectively (Table 5). Many factors may influence prediction accuracy [17,56]: marker density, training population size, the level of linkage disequilibrium in the population, the effective size of the population, the relatedness between the training and the test populations, the heritability of the trait, the architecture of the trait. In our plan of experiment, the fact that the training and test genotypes were phenotyped in the same environment probably over-estimated prediction accuracy. Similarly, sampling 2/3 of the population as training genotypes and 1/3 as test genotypes ensured genetic similarity between the training and test populations. On the other hand, the low density of markers and a moderate training population size may have limited prediction accuracy. Several authors [17,56] reported that decreasing marker number moderately affected prediction accuracy but that prediction accuracy increased linearly with training population size. Lorenz et al. [56] showed in genomic selection panels of barley, oat and wheat, that 300 SNP were sufficient to predict plant height and yield, with a training population size of 300. When the population size ranged from 100 to 300 lines, *Q*^2^ increased modestly and in the range of the values that we observed in our study. As noted by these authors, the training population size and marker density should scale together with effective population size. Hayes et al. [57] showed in barley, assuming an effective population size of 50 and a trait heritability of 1, that prediction accuracy reached ca. 0.45 with a training population size of 200 and ca. 0.9 for a training population size of 5000. The effective population size of the pea collection gathered here, which includes wild germplasm, is unknown but is probably larger than what would be observed in a breeding population. Depending on the architecture of traits, prediction methods may also impact prediction accuracy. In our study, the PLS, Bayes A, Bayes B, and GBLUP methods showed slightly better predictive efficiency than the SPLS and LASSO methods, except for NSeed. No method was the best in all cases. Similarly, Jannink et al. [17] reviewing dairy cattle Genomic Selection (GS) results observed that different models performed equally well. A few studies compared the efficiency of the different genomic prediction methods in crop plants. Iwata et al. [58] showed on simulated data that Ridge Regression was superior to a range of other methods including Bayes regression and PLS to predict phenotypes. However, Gouy et al. [59] and Resende et al. [60] did not find evidence, on sugarcane and loblolly pine data respectively, for any difference in the predictive ability of the different methods used (including BLUP, Bayes regression and LASSO). Our results also indicated that taking into account the structure as revealed by INSTRUCT and even more by DAPC did not improve the efficiency of phenotype prediction. We hypothesized that by subtracting part of the variability linked to population structure, we also reduced the genetic variance that can be explained by SNP.

Not surprisingly, the prediction efficiency was contrasted among the three traits considered. TSW, BegFlo, and Nseed are characterized by different heritability and genetic architecture. The genomic prediction proved the most useful for TSW (Table 5) that showed the highest broad-sense heritability and correlation among 2003 and 2007 values. The 2003 and 2007 field environmental conditions were contrasted, as regards to plant density and to climate: high temperatures (above 25°C) and water stress were encountered much earlier in 2007 than in 2003. Prediction coefficients were highly correlated for TSW. NSeed is less heritable and more subject to the environment than TSW and BegFlo, that are highly heritable traits in pea. Yet, the evolution of TSW and BegFlo may probably have significantly differed: flowering time may have been subject to local adaption depending on day length and climatic constraints. Jannink et al. [17] pointed out that while flowering time in maize may exhibit high heritabilities due to few loci controlling the trait, the genetic architecture of this trait may nonetheless be complex due to many variants clustered at these loci. To the contrary, seed weight has probably been subject to unidirectional selection towards bigger seeds since early domestication up to recent breeding. Burstin et al. [26] detected 7 genomic regions involved in BegFlo determination, 6 regions in TSW, and 3 regions in Nseed in one recombinant inbred line population. Burstin et al. [7] further located 77 QTL of thousand seed weight, corresponding to twenty-one metaQTL, in five recombinant inbred line populations involving parents from diverse origin. Among the SNP consistently involved in TSW prediction (agps2_SNP1,3; Hsp70_SNP1; RNAH_SNP4; At2g44950_SNP1, Bfruct_SNP1-4-7; ACCox_SNP3; CE007E18_SNP1; CE007H08_SNP1), 3 were located in the confidence interval of a TSW metaQTL, two were located near a metaQTL and the others were not mapped (Additional file 11). The level of linkage disequilibrium rapidly decreased with the genetic distance among markers in the collection (Figure 7). The list of SNP that were consistently associated with the prediction of TSW in the 367 accessions panel may thus tell us something about the molecular determinants of this trait. Pea seed is mainly composed of starch (ca 50%) and proteins (ca 23%). Seed weight is determined by the rate and the duration of seed growth [61], and associated with both the capacity of the seed to synthesize reserve and the capacity of the plant to provide the needed assimilates. Two of the TSW predictive SNP were found in genes involved in the starch and sucrose metabolism -Agps2 encoding an ADP-glucose-phosphorylase and bFruct a Beta-fructofuranosidase- and may be directly involved in pea seed size. Wrinkled seed phenotypes in pea have indeed been associated with incomplete seed filling and have been shown to be due to defect in starch accumulation [62]. For the other genes, a direct link with seed weight control is less straightforward. ACCox encodes for 1-aminocyclopropane-1-carboxylic acid oxidase, CE007H08 for a Galactinol synthase, Hsp70 for Heat shock protein Hsp70, RNAH for an ATP-dependent RNA helicase (RNAH), CE007E18 encodes a Hypothetical protein, and At2g44950 best homologue in Arabidopsis encodes a E3 ubiquitin ligase.

## Conclusion

This study confirmed the potential of different genotyping methods to reveal *Pisum* diversity at different evolutionary timescales. In spite of the limited number of SNP markers used in this study, these markers proved the most efficient in describing the genetic structure of our pea collection and showed promising results of genomic prediction for three phenotypic traits of interest. High-throughput SNP arrays that will soon be available will open the way to large scale genome-wide association studies as well as to new marker assisted applications in pea.

## Additional files

Additional file 1
**Table S1.** List of pea accessions used.

Additional file 2
**Table S2.** List of all markers used in this study and simple statistics of their polymorphism in the collection.

Additional file 3
**Figure S1.** Distribution of allele frequencies for all Simple Sequence Repeat (SSR) markers.

Additional file 4
**Figure S2.** Distribution of Minor Allele Frequency for Single Nucleotide Polymorphisms (SNP) and Retrotransposon-based Insertion Polymorphism (RBIP) markers.

Additional file 5
**Table S4.** Analysis of Variance of the Discriminant Analysis of Principal Component (DAPC) coordinates reveal the effect of use type, population type, sowing type and geographical origins.

Additional file 6
**Table S3.** Description of INSTRUCT and Discriminant Analysis of Principal Component (DAPC) groups, according to use type, population type and sowing type.

Additional file 7
**Table S5.** Average pairwise distances between accessions from the same or from different groups, as revealed by the DAPC and INSTRUCT procedure, for each of the 3 types of markers used.

Additional file 8
**Table S6.** List of the most predictive Single Nucleotide Polymorphisms (SNP) for Thousand Seed Weight (TSW), measured in 2003 and in 2007, for the different methods.

Additional file 9
**Figure S3.** Principal Component Analysis plots validated the ComBat procedure for population structure correction. Plots of the first two principal components before **(a and c)** and after **(b and d)** structure effect removal using ComBat: **(a and b)** for the INSTRUCT population structure (K =3) and **(c and d)** for the DAPC population structure (K =6).

Additional file 10
**Figure S4.** Plots of SNP prediction coefficients according to the Partial Least Square (PLS), Sparse Partial Least Squares (SPLS), and Least Absolute Shrinkage and Selection Operator (LASSO) methods, after taking or not into account the structure revealed by INSTRUCT and DAPC.

Additional file 11
**Figure S5.** Map positions of Thousand Seed Weight (TSW) consistently predictive SNP as compared to TSW MetaQTL according to [7].
