# Supplementary figures and images for: Genetic diversity and trait genomic prediction in a pea diversity panel
Source: BMC Genomics. 2015 Feb 21;16(1):105. doi: 10.1186/s12864-015-1266-1 (PMC4355348; doi:10.1186/s12864-015-1266-1)

## Slide 1
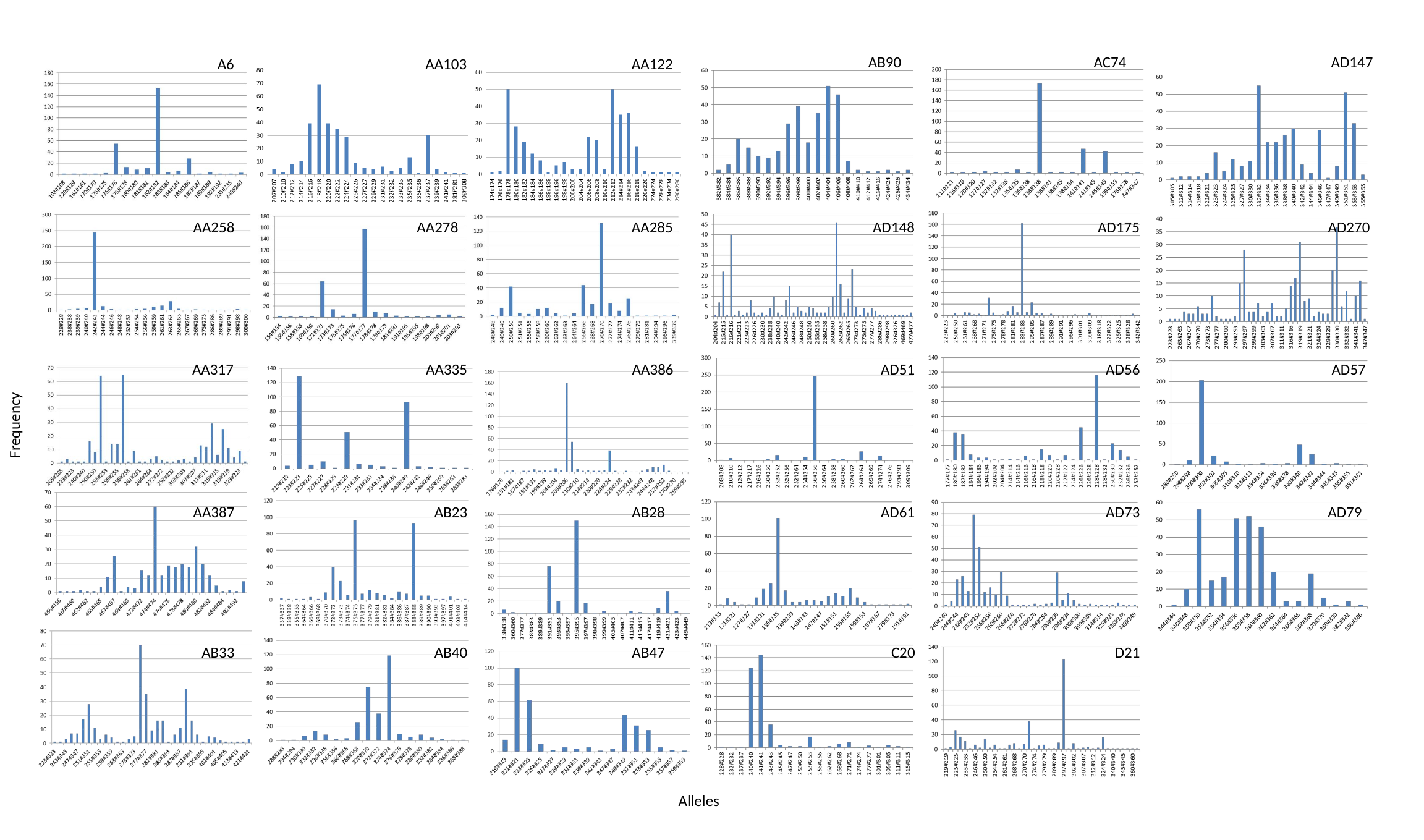

AB90
AC74
AD147
A6
AA103
AA122
AA258
AA278
AA285
AD148
AD175
AD270
AA317
AA335
AA386
AD51
AD56
AD57
Frequency
AA387
AB23
AB28
AD61
AD73
AD79
AB33
AB40
AB47
C20
D21
Alleles

Supplement: Additional file 3 — Figure S1. Distribution of allele frequencies for all Simple Sequence Repeat (SSR) markers. [file 12864_2015_1266_MOESM3_ESM.pptx]

## Slide 1
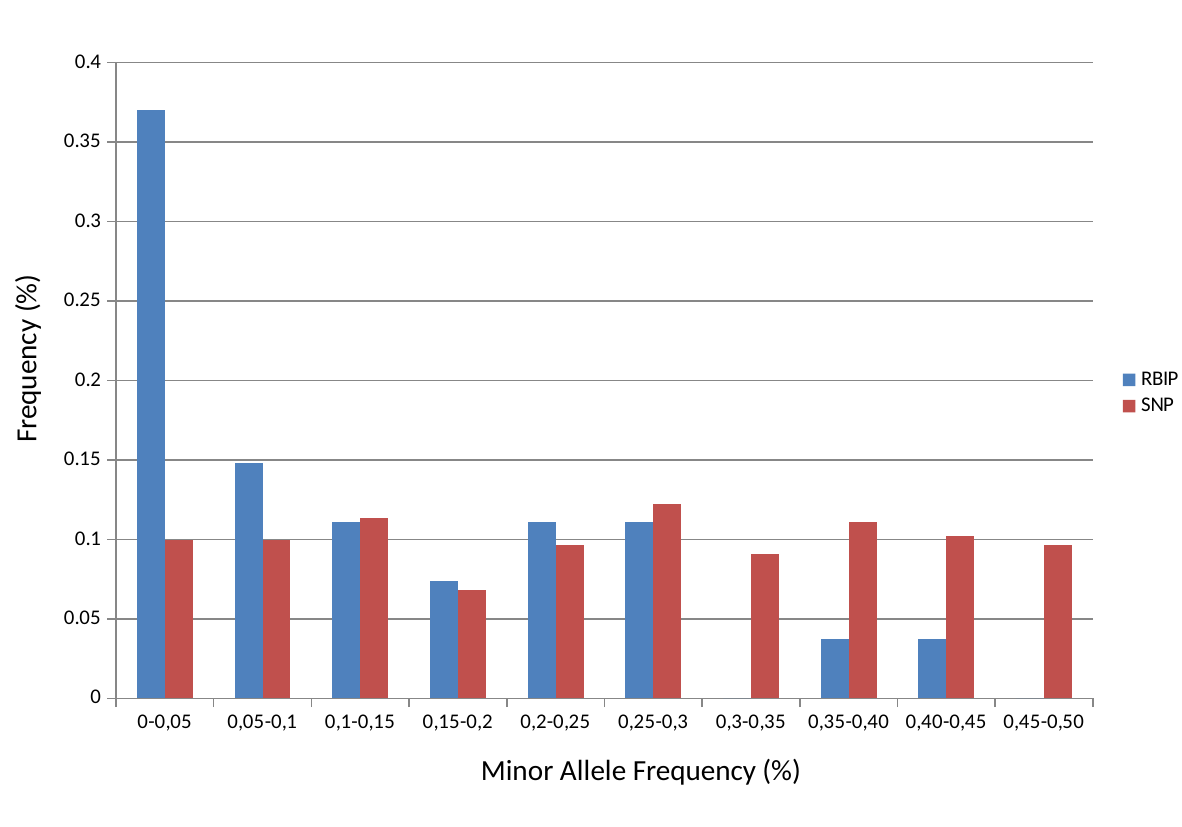

### Chart
| Category | RBIP | SNP |
|---|---|---|
| 0-0,05 | 0.3703703703703707 | 0.09943181818181823 |
| 0,05-0,1 | 0.14814814814814825 | 0.09943181818181823 |
| 0,1-0,15 | 0.11111111111111112 | 0.1136363636363636 |
| 0,15-0,2 | 0.07407407407407408 | 0.06818181818181818 |
| 0,2-0,25 | 0.11111111111111112 | 0.09659090909090916 |
| 0,25-0,3 | 0.11111111111111112 | 0.12215909090909091 |
| 0,3-0,35 | 0.0 | 0.09090909090909102 |
| 0,35-0,40 | 0.037037037037037056 | 0.1107954545454546 |
| 0,40-0,45 | 0.037037037037037056 | 0.10227272727272729 |
| 0,45-0,50 | 0.0 | 0.09659090909090916 |Frequency (%)
Minor Allele Frequency (%)

Supplement: Additional file 4 — Figure S2. Distribution of Minor Allele Frequency for Single Nucleotide Polymorphisms (SNP) and Retrotransposon-based Insertion Polymorphism (RBIP) markers. [file 12864_2015_1266_MOESM4_ESM.pptx]

## Slide 1
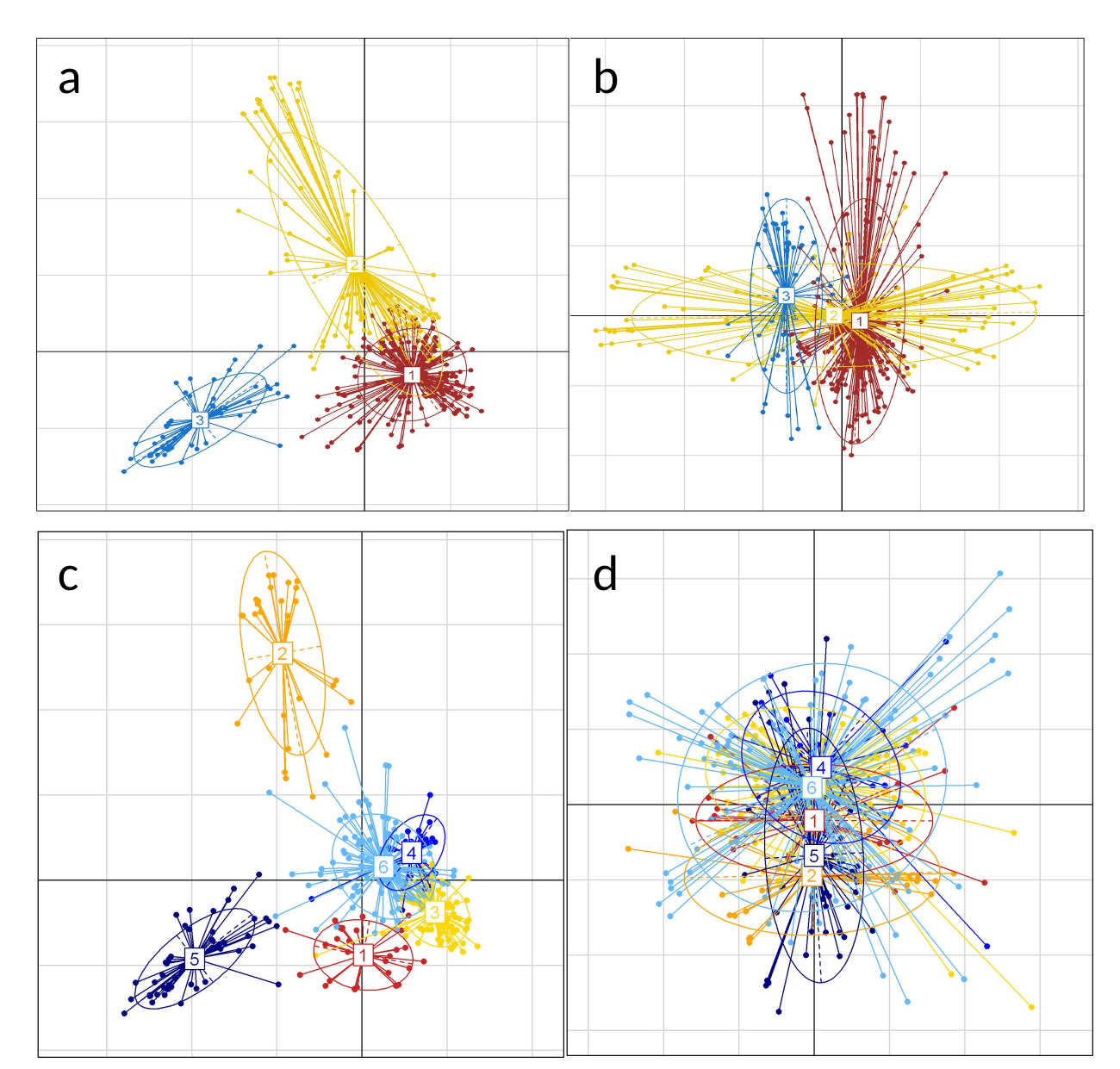

a
b
c
d

Supplement: Additional file 9 — Figure S3. Principal Component Analysis plots validated the ComBat procedure for population structure correction. Plots of the first two principal components before (a and c) and after (b and d) structure effect removal using ComBat: (a and b) for the INSTRUCT population structure (K =3) and (c and d) for the DAPC population structure (K =6). [file 12864_2015_1266_MOESM9_ESM.pptx]

## Slide 1
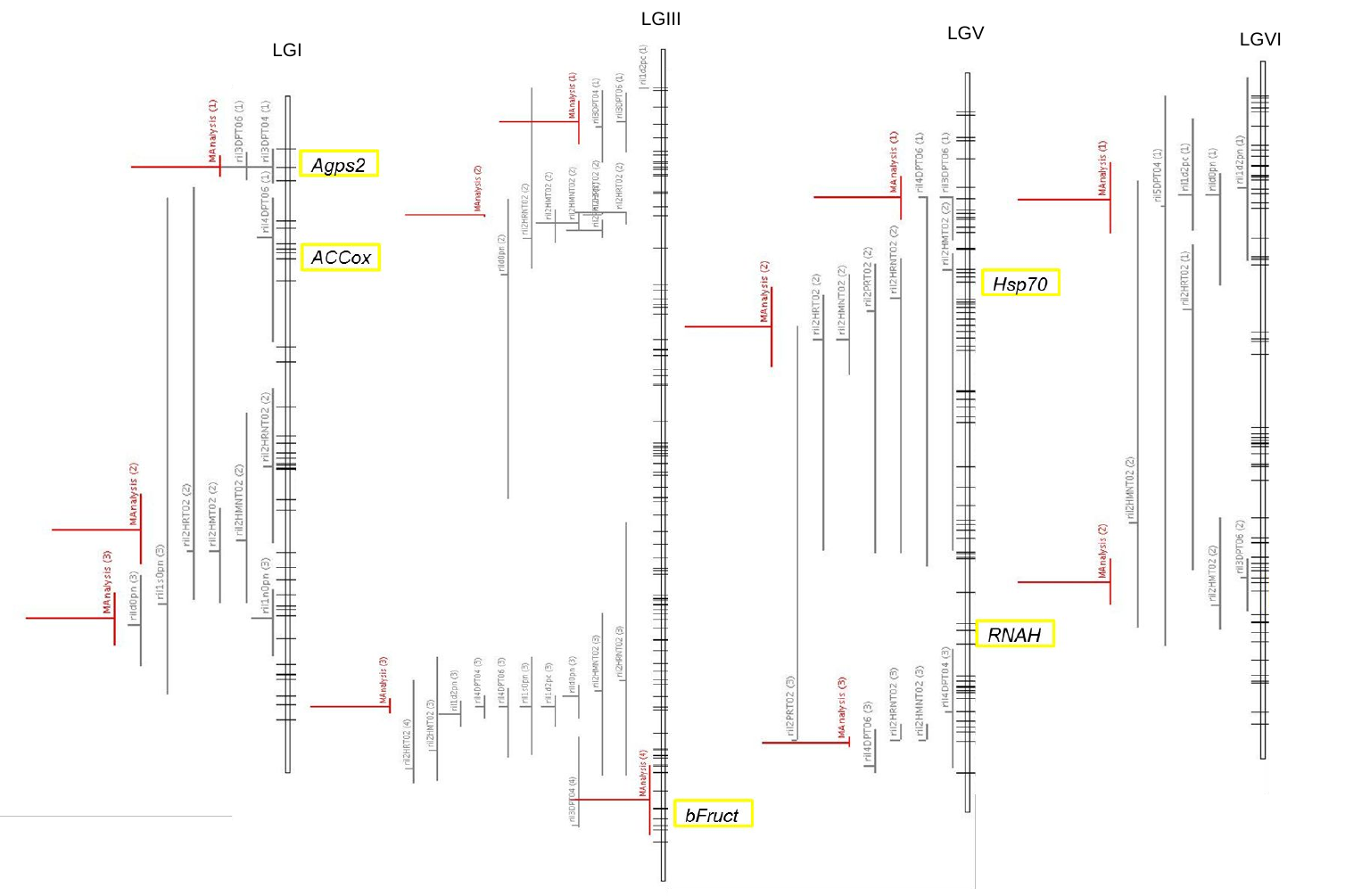

LGIII
LGV
LGVI
LGI

Supplement: Additional file 11 — Figure S5. Map positions of Thousand Seed Weight (TSW) consistently predictive SNP as compared to TSW MetaQTL according to [7]. [file 12864_2015_1266_MOESM11_ESM.pptx]
