# Supplementary material for: Genetic diversity and trait genomic prediction in a pea diversity panel
Source: BMC Genomics. 2015 Feb 21;16(1):105. doi: 10.1186/s12864-015-1266-1 (PMC4355348; doi:10.1186/s12864-015-1266-1)
Supplement: Additional file 10 — Figure S4. Plots of SNP prediction coefficients according to the Partial Least Square (PLS), Sparse Partial Least Squares (SPLS), and Least Absolute Shrinkage and Selection Operator (LASSO) methods, after taking or not into account the structure revealed by INSTRUCT and DAPC. [file 12864_2015_1266_MOESM10_ESM.pptx]

## Slide 1
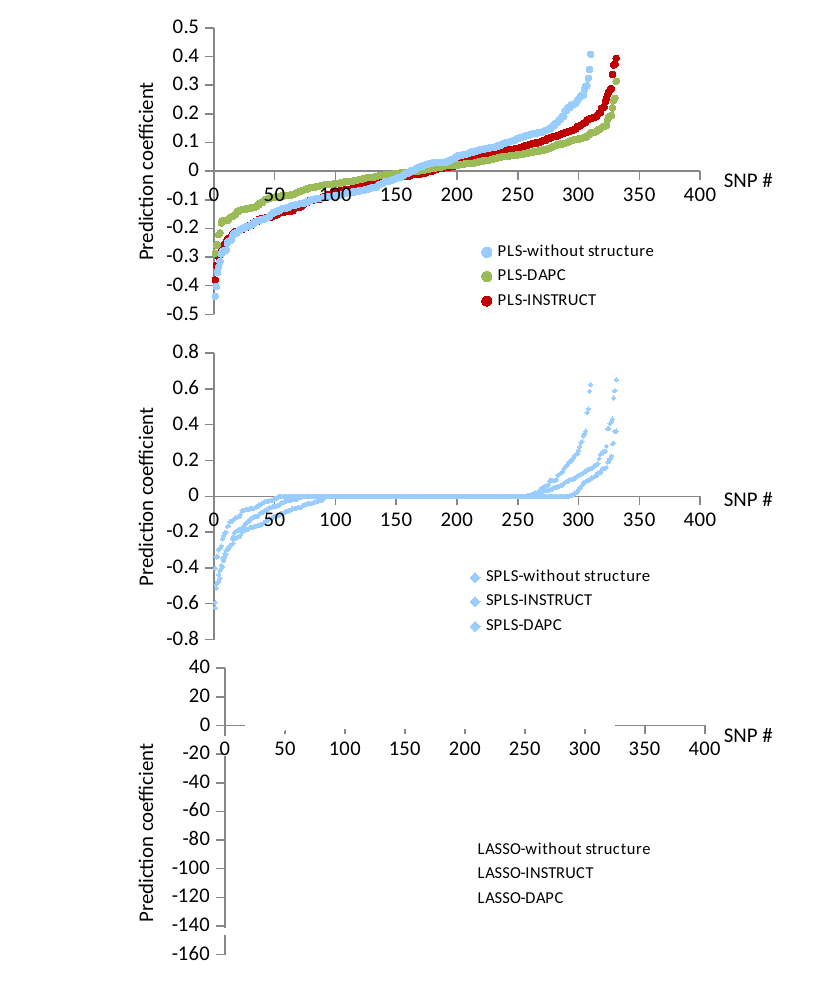

### Chart
| Category | PLS-without structure | PLS-DAPC | PLS-INSTRUCT |
|---|---|---|---|Prediction coefficient
SNP #
### Chart
| Category | SPLS-without structure | SPLS-INSTRUCT | SPLS-DAPC |
|---|---|---|---|Prediction coefficient
SNP #
### Chart
| Category | LASSO-without structure | LASSO-INSTRUCT | LASSO-DAPC |
|---|---|---|---|SNP #
Prediction coefficient
